# Structure of eukaryotic DNA polymerase δ bound to the PCNA clamp while encircling DNA

**DOI:** 10.1073/pnas.2017637117

**Published:** 2020-11-17

**Authors:** Fengwei Zheng, Roxana E. Georgescu, Huilin Li, Michael E. O’Donnell

**Affiliations:** ^a^Structural Biology Program, Van Andel Institute, Grand Rapids, MI 49503;; ^b^DNA Replication Laboratory, The Rockefeller University, New York, NY 10065;; ^c^HHMI, The Rockefeller University, New York, NY 10065

**Keywords:** DNA polymerase, sliding clamp, PCNA, DNA polymerase δ, DNA replication

## Abstract

The DNA polymerase (Pol) δ of *Saccharomyces cerevisiae* (S.c.) is composed of the catalytic subunit Pol3 along with two regulatory subunits, Pol31 and Pol32. Pol δ binds to proliferating cell nuclear antigen (PCNA) and functions in genome replication, repair, and recombination. Unique among DNA polymerases, the Pol3 catalytic subunit contains a 4Fe-4S cluster that may sense the cellular redox state. Here we report the 3.2-Å cryo-EM structure of S.c. Pol δ in complex with primed DNA, an incoming ddTTP, and the PCNA clamp. Unexpectedly, Pol δ binds only one subunit of the PCNA trimer. This singular yet extensive interaction holds DNA such that the 2-nm-wide DNA threads through the center of the 3-nm interior channel of the clamp without directly contacting the protein. Thus, a water-mediated clamp and DNA interface enables the PCNA clamp to “waterskate” along the duplex with minimum drag. Pol31 and Pol32 are positioned off to the side of the catalytic Pol3-PCNA-DNA axis. We show here that Pol31-Pol32 binds single-stranded DNA that we propose underlies polymerase recycling during lagging strand synthesis, in analogy to *Escherichia coli* replicase. Interestingly, the 4Fe-4S cluster in the C-terminal CysB domain of Pol3 forms the central interface to Pol31-Pol32, and this strategic location may explain the regulation of the oxidation state on Pol δ activity, possibly useful during cellular oxidative stress. Importantly, human cancer and other disease mutations map to nearly every domain of Pol3, suggesting that all aspects of Pol δ replication are important to human health and disease.

## Significance

The structure of the eukaryotic chromosomal replicase, DNA polymerase (Pol) δ, was determined in complex with its cognate proliferating cell nuclear antigen (PCNA) sliding clamp on primed DNA. The results show that the Pol3 catalytic subunit binds atop the PCNA ring, and the two regulatory subunits of Pol δ, Pol31, and Pol32, are positioned off to the side of the Pol3 clamp. The catalytic Pol3 binds DNA and PCNA such as to thread the DNA straight through the circular PCNA clamp. Considering the large diameter of the PCNA clamp, there is room for water between DNA and the inner walls of PCNA, indicating the clamp “waterskates” on DNA during function with polymerase.

Replication of the cellular DNA genome is accomplished by DNA polymerases (Pol) that function with a ring shaped “sliding clamp” (1–3). The sliding clamp encircles duplex DNA and tethers the Pol to DNA for high processivity during synthesis (4, 5). Sliding clamps are assembled onto primed-template (T/P) junctions by a pentameric clamp loader that couples ATP binding and hydrolysis to opening and closing of the ring around DNA (6). All three domains of life utilize an evolutionarily conserved sliding clamp and clamp loader; in eukaryotes/archaea, these are the proliferating cell nuclear antigen (PCNA) clamp and replication factor C (RFC), and in bacteria they are referred to as the β clamp and γ complex (7). DNA polymerases assort into seven families by sequence homology—families A, B, C, D, X, Y, and reverse transcriptase—although structural analysis shows that all DNA polymerases are shaped like a right hand and contain subdomains referred to as palm, fingers, and thumb (8). The cellular replicative polymerases of the three domains of life belong to the C family (bacteria), the B family (eukaryotes), or the B and D family (archaea).

Eukaryotes utilize three essential B family DNA polymerases for genome replication: Pol α, Pol δ, and Pol ε (9). Each of these B family DNA polymerases is highly conserved from yeast to human (9). Pol α, referred to as Pol α-primase, generates 20- to 30-nt-long RNA-DNA primers to initiate the synthesis of leading and lagging strands. Pols δ and ε are the main replicative polymerases, and each contain a proofreading 3′-5′ exonuclease. Pol ε replicates the bulk of the leading strand (9, 10) and functions optimally with the replicative CMG helicase to which it binds (11, 12). Pol δ performs lagging strand synthesis by extending primers generated by Pol α primase every 100 to 200 nt (9). Pol δ also synthesizes a small amount of leading strand DNA during replication initiation and termination (13, 14). Both Pol δ and Pol ε function with the PCNA sliding clamp (9, 15). In fact, Pol δ has little activity in the absence of PCNA, which stimulates Pol δ activity by a factor of 30 (16).

The sliding clamps of bacteria and eukaryotes are structurally superimposable and are also utilized in numerous processes beyond replication, including use by translesion synthesis (TLS) Pols, and a multitude of different repair factors, such as DNA ligase, Fen1 nuclease, MutS, and MutL to mention only a few, making sliding clamps an attractive therapeutic target (15, 17). Sliding clamps are homo-oligomers, and each subunit contains a hydrophobic pocket to which proteins attach (1, 17). Thus, clamps have the potential to simultaneously bind multiple partners, acting like a “tool belt.” In eukaryotes, the motif that proteins use to attach to the clamp’s hydrophobic pocket is referred to as a PCNA interaction peptide (PIP) motif, and a similar motif is utilized by proteins that attach to the bacterial β clamp (18). The canonical PIP motif is QxxΨxxθθ (where Ψ is hydrophobic, θ is aromatic, and x is any residue). There are also noncanonical PIP sequences that differ widely from the canonical PIP motif (19).

This report focuses on the *Saccharomyces cerevisiae* (S.c.) replicative Pol δ and its interaction with the PCNA clamp. S.c. Pol δ contains three subunits. Pol3 harbors the catalytic Pol and proofreading 3′-5′ exonuclease, and the regulatory subunits are Pol31 (i.e., the B subunit) and Pol32. Human Pol δ contains an additional regulatory subunit, Pol12 (9). The exact functions of the “regulatory subunits” are not well defined. Unique to DNA polymerases, the catalytic Pol3 subunit of Pol δ contains a 4Fe-4S iron sulfur cluster (20). During lagging strand synthesis, Pol δ is capable of releasing its PCNA clamp soon after completing synthesis of a DNA gap (i.e., Okazaki fragment) (21), similar to observations in the *Escherichia coli* replicase system (22, 23). In addition, Pol δ-PCNA functions with the Fen1 5′ nuclease on a millisecond time scale to strand-displace and excise the 5′ RNA-DNA of Okazaki fragments, followed by DNA ligase I (Lig1) that seals the nick (16). This rapid timescale suggests that Fen1 and Lig1 bind PCNA simultaneously with Pol δ for this frequent Okazaki fragment maturation reaction. Indeed, an archaeal PCNA is a heterotrimer in which each clamp subunit binds a separate factor, Pol D, Fen1, or ligase. Indeed, PCNA in the archaeon *Sulfolobus solfataricus* is a heterotrimer in which each clamp subunit binds a separate factor: Pol B, Fen1, or ligase (24, 25).

Pol δ has a far greater role beyond its role in DNA replication. For example, Pol δ, along with Pol η, is required for lesion bypass on both leading- and lagging-strand DNA (26). Pol δ-PCNA functions with other proteins to perform break-induced repair, which can proceed for many thousands of nucleotides (27). Pol δ is also required during meiotic recombination to extend 3′ ends that are exchanged among homologous chromosomes, holding them together during the first cell division (28). The regulatory subunits, Pol31 and Pol32, are also shared with the TLS Pol ζ (29). The exact reason why the Pol31 and Pol32 subunits are shared among Pol δ and Pol ζ remains a mystery.

The structures of yeast Pol δ-DNA and human Pol δ-PCNA-DNA have been reported while the present study was in progress (30, 31). In this work, we examine S.c. Pol δ-PCNA-DNA and compare and contrast the earlier findings with our present results, and also note some relevant comparisons with archaeal Pol D-PCNA (32) and *E. coli* Pol III-β clamp-DNA (33). Overall, the results provide a unifying view of the structure of replicative polymerase-clamp complexes and the mechanism by which sliding clamps move along DNA during replication, and provide insight into possible roles of Pol31, Pol32, and regulation of Pol δ by the oxidation-reduction state of the cell.

## Results

### Overall Structure of the S.c. Pol δ-PCNA-DNA-ddTTP Complex.

**Fig. 1.**
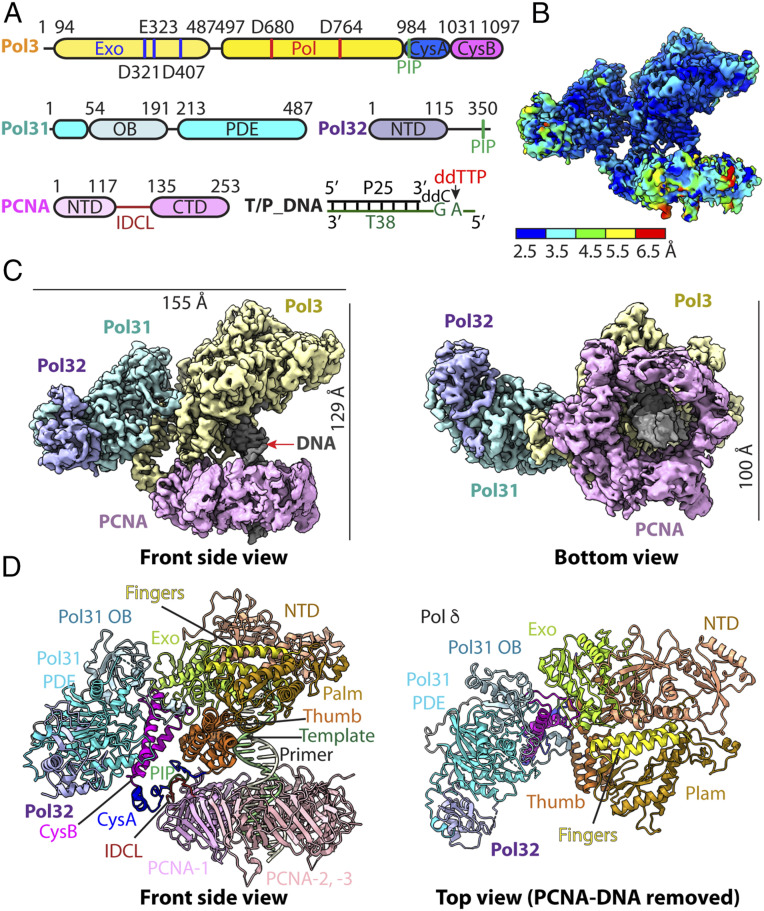
Cryo-EM structure of the S.c. Pol δ-PCNA-DNA ternary complex. (*A*) Domain architecture of Pol δ subunits Pol3, Pol31, and Pol32. Pol3 exonuclease active site residues D321, E323, and D407 and polymerase active site residues D608 and D704 are labeled. The DNA substrate is also sketched. (*B*) Local resolution estimation of the cryo-EM 3D map. (*C*) 3D map segmented by subunits and colored individually in a side and a bottom view. (*D*) Cartoon view of the atomic model of Pol δ-PCNA-DNA in the side and top views. PCNA and DNA are removed on the right for a clear view of Pol δ.

The holoenzyme of S.c. Pol δ-PCNA-DNA-ddTTP was assembled in vitro by directly mixing purified S.c. Pol δ, PCNA, a primed template (25 nt primer, 38 nt template strand; hereinafter referred to as T/P DNA), and ddTTP (Fig. 1*A*). We introduced two mutations, D321S and E323S, into the proofreading 3′-5′ exonuclease active site of Pol3 to prevent degradation of the T/P DNA. By two-dimensional (2D) and three-dimensional (3D) classifications of the cryo-EM images, we found ∼8.4% of the particle population assembled into the full complex. The 3D reconstruction and refinement of the subdataset containing some 430,000 holoenzyme particles led to a 3D map at 3.2-Å resolution and the building of the first atomic model of the S.c. Pol δ holoenzyme (Fig. 1 *B* and *C* and *SI Appendix*, Fig. S1 and Table S1. Many amino acid side chains could be seen to fit nicely into the EM map (*SI Appendix*, Fig. S2). This complex has physical dimensions of 155 Å × 100 Å × 129 Å (Fig. 1*C*).

The structure shows that Pol3 is the only subunit of Pol δ that binds PCNA, mediated through a single subunit of the PCNA trimer (Fig. 1 *C* and *D* and Movie S1). This single interface with PCNA contrasts with the expected attachment of all three Pol δ subunits to PCNA implied in an earlier study (34). Interestingly, the regulatory subunits, Pol31 and Pol32, are oriented laterally with respect to the Pol3-PCNA-DNA axis and do not interact with DNA (Fig. 1 *C* and *D*). However, we note that Pol31 contains an oligosaccharyl/oligonucleotide binding (OB) fold and an inactive phosphodiesterase and polymerase domain (PDE), sometimes referred to as a polymerase and histidinol phosphatase (PHP) domain, which might interact with downstream single-strand (ss) DNA (Fig. 1 *C* and *D*). Indeed, we found that isolated Pol31-Pol32 bound to ssDNA in electrophoretic mobility shift assays (EMSAs) (*SI Appendix*, Fig. S3). The PDE/PHP domain is also present in bacterial Pol III and is an active proofreading 3′-5′ exonuclease in some Pol IIIs while being inactive in other Pol IIIs that have recruited a separate 3′-5′ exonuclease subunit/domain (35). Active PDE/PHE domains consist of several α helices in a barrel conformation and harbor catalytic metals coordinated by nine conserved residues; the inactive *E. coli* Pol III PDE/PHP domain has lost five metal-binding residues during evolution (*SI Appendix*, Fig. S4 *A* and *B*). The PDE/PHP domain of Pol δ has lost six metal binding residues, and the barrel shape is less compact and somewhat distorted. However, a human disease mutation maps to this location, suggesting the PDE/PHP domain is important to Pol δ function (*SI Appendix*, Table S2).

While this work was ongoing, the structure of human Pol δ-PCNA-DNA was reported (32); it has the same overall features as the S.c. Pol δ-PCNA-DNA structure, with an RMSD_Cα_ of ∼2.9 Å (*SI Appendix*, Fig. S5 *A* and *B*). Thus, both structures adopt an arrow-shaped architecture, with the catalytic Pol3 subunit sitting on the proximal face of PCNA and the regulatory subunits angled sideways from the Pol3-PCNA-DNA axis.

### The 4Fe-4S Cluster Occupies a Central Position in the Pol δ-PCNA-DNA Architecture.

**Fig. 2.**
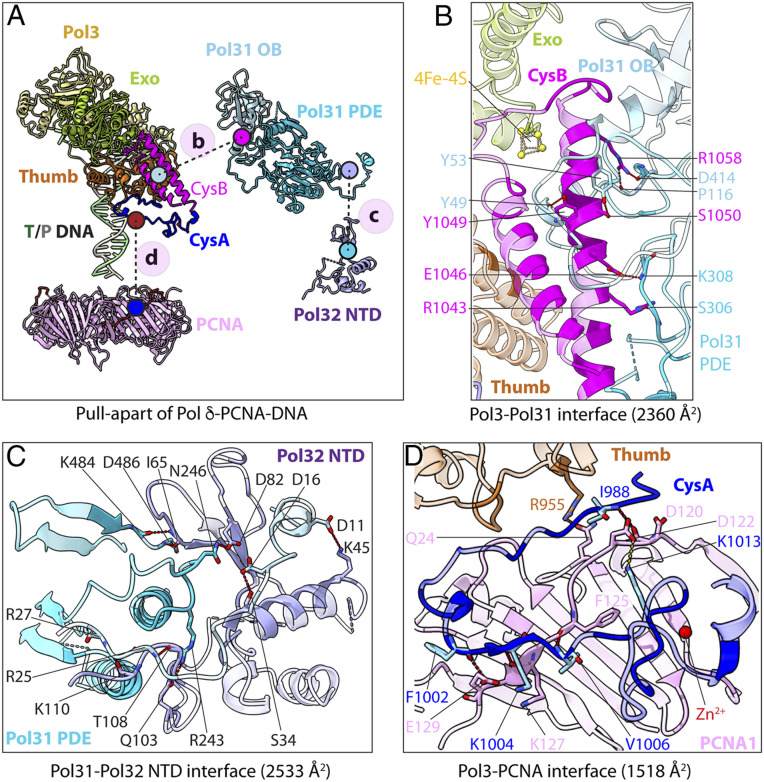
Subunit-subunit interfaces in Pol δ-PCNA-DNA. (*A*) Pull-apart of the Pol δ-PCNA-DNA structure to show the three major subunit-subunit interfaces between Pol3 and Pol31 (*B*), between Pol31 and Pol32 (*C*), and between Pol3 and PCNA (*D*). (*B*–*D*) Close-up views of the three subunit-subunit interfaces with key residues labeled. The side chains of Y49 and K308 are omitted for clarity.

Pol δ is the only DNA polymerase known to contain a 4Fe-4S cluster, and its role remains uncertain. Oxidation of the 4Fe-4S cluster reportedly slows DNA synthesis by Pol δ, suggesting a possible regulatory role of the cluster (*Discussion*) (20). The protein connections within the Pol δ-PCNA-DNA complex show a linear progression of three binary subunit-subunit contacts that result in a curved architecture: PCNA to Pol3, Pol3 to Pol31, and Pol31 to Pol32 (Fig. 2). The C-terminal CysB element in Pol3 contains the 4Fe-4S cluster coordinated by 4 Cys residues of two antiparallel α-helices (*SI Appendix*, Fig. S6*A*). The 4Fe-4S coordinating element of Pol3 is much smaller than that of the Pri2 subunit of the priming apparatus within Pol α-primase, which is composed of several secondary structure elements (*SI Appendix*, Fig. S6*A*).

**Fig. 3.**
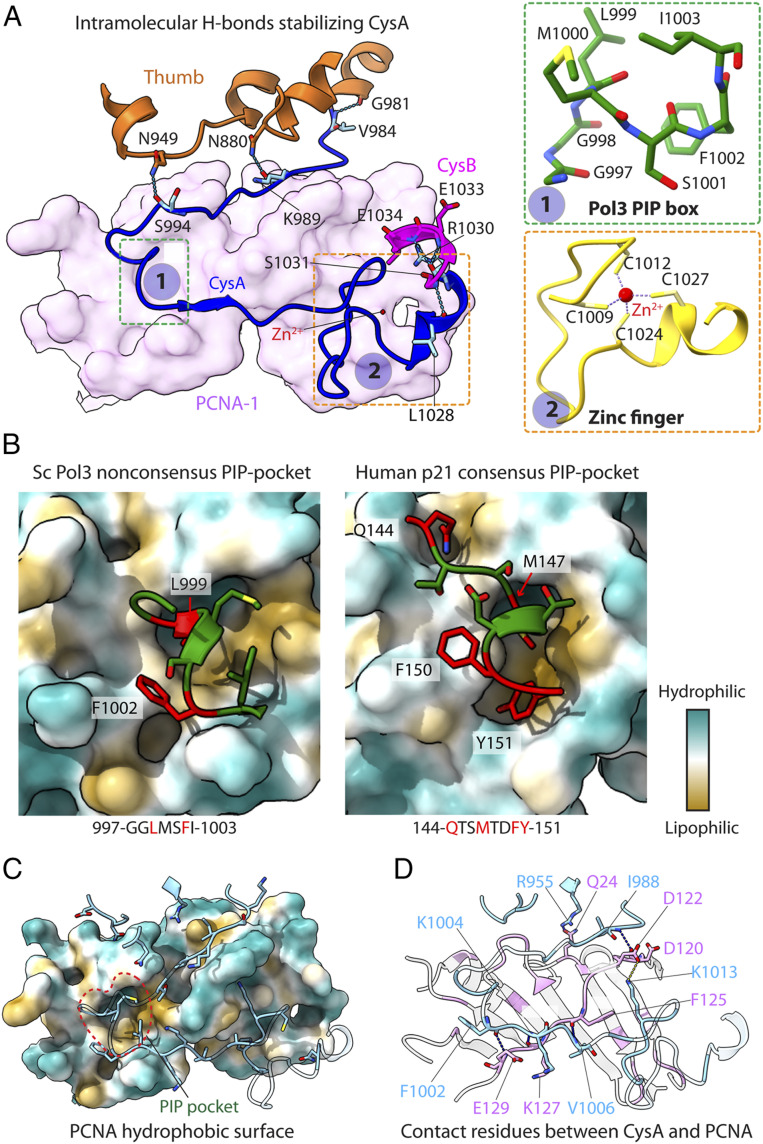
Pol3 CysA is stabilized by intramolecular and intermolecular interactions. (*A*) Intramolecular interactions between the Pol3 thumb domain and CysA and CysB motifs. PCNA is in pink surface, CysA in blue, and thumb domain is in orange cartoon. (*Inset 1*) The CysA PIP in green sticks. (*Inset 2*) The zinc finger in yellow cartoon. (*B*) The interaction of the CysA nonconsensus PIP motif (*Left*) and a representative consensus p21 PIP motif (PDB ID code 1AXC; *Right*) with the hydrophobic pocket of PCNA in surface view colored by hydrophobicity. (*C*) Interaction of the Pol3 CysA N-terminal peptide nests in a hydrophobic groove next to the IDCL of PCNA. Residues involved in the interactions are shown. (*D*) The contact residues between CysA and PCNA. Residues involved in the hydrogen bonds (dashed cyan lines) and salt bridge (dashed yellow line) are labeled.

Importantly, the 4Fe-4S cluster is centrally located in the holoenzyme and functions to glue together the Pol3 and the regulatory Pol31 and Pol32 subunits by interacting with the OB and PDE elements of Pol31, resulting in an extensive interface of 2,360 Å^2^ (Fig. 2 *A* and *B*). This is also noted in the S.c. Pol δ structure in the absence of PCNA (30). Next, Pol31 forms a sizeable interface of 2,533 Å^2^ with Pol32 (Fig. 2*C*). Interaction with PCNA is mediated by the C-terminal CysA element of Pol3 that immediately precedes CysB in the primary sequence and is adjacent to CysB in the structure, and might be affected by the oxidation state of the 4Fe-4S cluster. CysA contains a zinc finger and a nonconsensus PIP site, both of which are involved in binding a single protomer of the trimeric PCNA ring with a smaller interface of 1,518 Å^2^ (Fig. 2*D*), accounting for the partial flexibility of the PCNA ring (Figs. 2 and 3), as described further below.

### Interaction of Pol δ with PCNA.

The EM map of Pol δ-PCNA-DNA shows 22 bp of ordered dsDNA extending from the Pol3 active site and through PCNA (Figs. 1*D* and 2*A*). This stands in contrast to the 12-bp dsDNA in the Pol δ-DNA structure (i.e., lacking PCNA) (30), indicating that the PCNA ring orders the additional dsDNA. In our structure, as in the recently described human Pol δ-PCNA-DNA structure (32), only one half of the PCNA ring on the Pol δ-PCNA binding side had strong density; the other half was somewhat flexible and only had helix densities in the inner layer of the PCNA ring. The atomic model of the PCNA in the weak-density region was built by rigid body docking. The entire CysA element (residues 985 to 1,029) is disordered in the S.c. Pol δ-DNA structure lacking PCNA (30) but becomes ordered in our S.c. Pol δ-PCNA-DNA structure on binding of PCNA.

The CysA element contains a nonconsensus PIP motif (997-GGLMSFI-1003) that is substantially distinct from the consensus PIP motif (19), as well as a zinc finger motif (amino acids 1,009 to 1,030) with its four cysteine residues (C1009, C1012, C1024, and C1027) coordinating the zinc ion (Zn^2+^) (Fig. 3*A*). CysA is stabilized by both intramolecular and intermolecular interactions (Fig. 3 *A*–*D*). The CysA motif forms six intramolecular H-bonds, with V984, K989, S994, L1028, and R1030 H-bonding with G981, N880, N949, S1031, E1033, and E1034 of the thumb domain, respectively (Fig. 3*A*). CysA R1030 interacts with both CysB E1033 and E1034. The intermolecular interface involves both hydrophobic and hydrophilic interactions (Fig. 3 *B*–*D*). The N-terminal peptide of CysA (amino acids 984 to 1,003) is nested in the shallow hydrophobic groove lined by the interdomain connecting loop (IDCL) of PCNA (Fig. 3*C*). In fact, the CysA peptide and the PCNA IDCL pair up to form a two-stranded β-sheet (Fig. 3 *C* and *D* and *SI Appendix*, Fig. S6*B*). Interestingly, the p21 peptide following the consensus PIP motif also forms a two stranded β-sheet, which is much longer than the CysA-IDCL β-sheet (*SI Appendix*, Fig. S6*B*). At the terminus of this shallow groove is the hydrophobic PIP-binding pocket. The Pol3 nonconsensus PIP motif plugs into this pocket but does not extend further into the shallower part of the hydrophobic groove on PCNA, which is used by the consensus PIP sequences, as illustrated by a comparison with the consensus PIP binding of human p21 (Fig. 3*B*). An extended comparison of the nonconsensus PIP binding (RFC1, S.c. Pol3, human P125) vs. the consensus PIP binding (Fen1, p21) appears to validate this generalization (*SI Appendix*, Fig. S6*C*). The intermolecular hydrophilic interactions that order the CysA motif consist of five H-bonds between Pol3 and PCNA (R995-Q24, I988-D120, F1002-E129, K1004-K127, and V1006-F125) and one salt bridge (K1013-D122) (Fig. 3*D*).

In total, the interface of Pol3 CysA with PCNA involves 27 Pol3 residues and 28 PCNA residues with an area of 1,518 Å^2^ (Fig. 2*D*). Interestingly, the interface of human Pol3-PCNA involves only 21 p125 residues and 21 PCNA residues with an area of 1,022 Å^2^ (*SI Appendix*, Fig. S7). This leads to an ∼50% larger yeast Pol δ-PCNA interface compared with the interface between human Pol δ and PCNA. This large interaction area likely underlies the observed greater processivity of yeast Pol δ-PCNA (21) compared with human Pol δ-PCNA (36). The biological reason for the significantly different processivities in yeast and human Pol δ-PCNA is not clear at this time.

### DNA–Protein Interactions in Pol δ-PCNA-DNA.

**Fig. 4.**
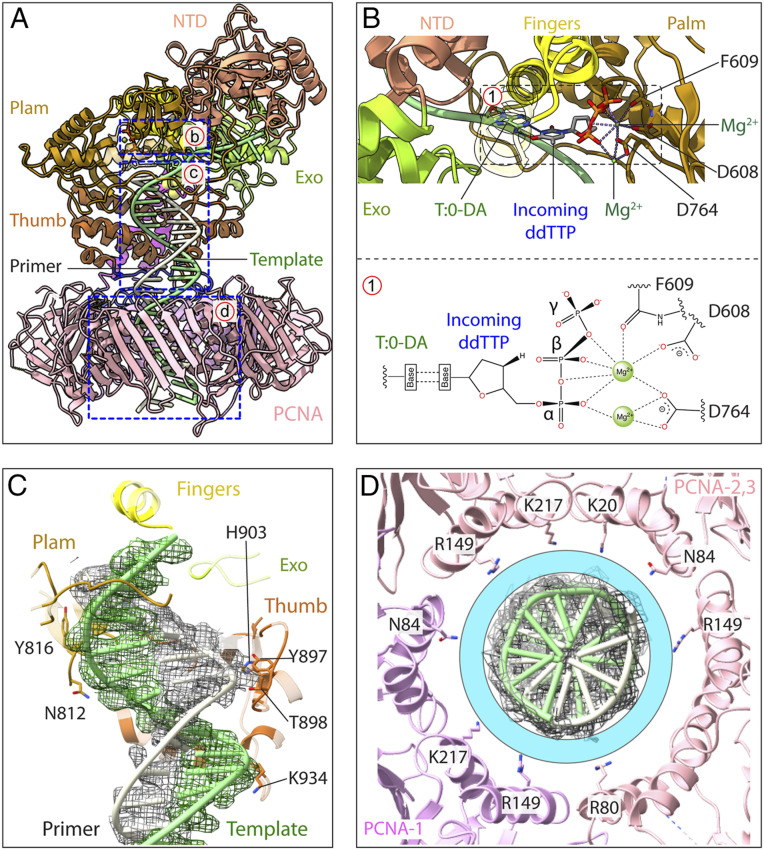
Protein–DNA interactions in Pol δ-PCNA-DNA. (*A*) Structure of Pol3-DNA on PCNA with Pol31 and Pol32 removed. The regions in the three dashed blue boxes highlight protein–DNA interactions that are magnified in *B*–*D*. (*B*) The Pol3 active site. D764 and D608 in the palm coordinate two Mg^2+^ ions. The incoming ddTTP is coordinated by the two Mg^2+^ ions and is base-paired with a template dA (T:0-DA). (*C*) The Pol3 residues that interact with the DNA. (*D*) The 6-Å circular cyan band marks the space between dsDNA and the inner surface of the PCNA ring. This band is filled with a layer of water molecules.

The yeast Pol δ-PCNA-DNA-ddTTP structure is frozen at a stage at which the incoming ddTTP forms a Watson–Crick base pair with the template strand but is not incorporated into the primer because the last primer nucleotide is the dideoxyribonucleotide ddG (Fig. 4 *A*–*D*). Within the catalytic pocket of Pol3, the palm (N812 and Y816), thumb (Y897, T898, H903, and K934), and fingers subdomains interact mainly with the template strand of the T/P junction (Fig. 4*C*). There are three conserved acidic residues (D608, D762, and D764) in the palm domain, two of which coordinate the two Mg^2+^ ions (residues D608 and D764) that in turn interact with the phosphodiester bonds of the incoming ddTTP (Fig. 4*B*). Activation of the 3′ ribose hydroxyl by Mg^2+^ is not observed here because it does not exist in the last primer nucleotide, which is a 2′ and 3′ dideoxynucleotide.

Most interestingly, in the presence of Pol δ, the DNA is held at a nearly perpendicular angle (84°) to the plane of PCNA, and there appears to be no distortion of the B-form DNA (Figs. 1 *C* and *D* and 4 *A* and *C*, *SI Appendix*, Fig. S5, and Movie S1). This nearly perpendicular arrangement enables basic side chains on the interior surface of the PCNA ring to reach toward the phosphate backbone, but they do not appear to directly contact the DNA. For example, N84, R149, and K217 of PCNA are held >6 Å away from the DNA phosphate backbone (Fig. 4*D* and Movie S1). This suggests that a single layer of water molecules is accommodated between the inner surface of PCNA and the DNA, which has implications for the process by which the PCNA ring slides on DNA (*Discussion*). This perpendicular orientation is in sharp contrast to the 62° angle of DNA through PCNA in the absence of Pol δ, which enables multiple direct contacts of PCNA to DNA (*SI Appendix*, Fig. S5), and these contacts may serve a separate purpose (*Discussion*) (36).

### Cancer Mutations in Pol δ-PCNA.

**Fig. 5.**
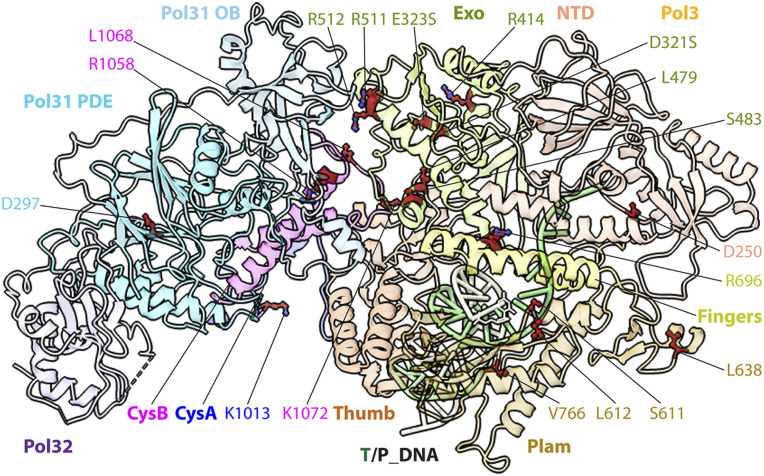
Reported disease-causing mutations in human Pol δ mapped onto the S.c. Pol δ structure. These mutations are associated with various diseases (*SI Appendix*, Table S2). The mutations are distributed on all the domains of catalytic subunit Pol3 except the thumb domain. Residue K1013 (human R1016) is located in the Pol3-PCNA interface and forms a salt bridge with PCNA D122 in both the yeast and human Pol δ-PCNA-DNA structures. There is also one mutation (D297, human D293) in the Pol31 legacy nuclease PDE domain.

Mutations in the exonuclease and other regions of Pol δ are known to be associated with colon cancer and other diseases (37–41). Consulting the human disease genetic databases, we mapped several known disease-causing mutations on S.c. Pol δ (Fig. 5 and *SI Appendix*, Table S2). The mutations mapped to essentially all the major domains within Pol3, including the CysB element that forges the interface to the regulatory subunits. One mutation even mapped to the Pol3-PCNA interface. Interestingly, one disease mutation mapped in the Pol31 PDE domain, the legacy nuclease domain that lost its nucleolytic function during evolution. Thus, it seems possible that the Pol31 PDE may have acquired a new role, rather than simply being a relic of evolution. In summary, the numerous disease mutations that map to nearly all domains of Pol δ suggest the importance to disease of essentially all aspects, known and unknown, of Pol δ action.

## Discussion

### Structure of Pol δ-PCNA-DNA.

In this study, we determined the structure of the S.c. replicative Pol δ-PCNA-DNA. Unexpectedly, we found that the regulatory subunits of Pol δ (Pol31 and Pol32) are positioned laterally, off to the side of the Pol δ-PCNA-DNA axis. Surprisingly, Pol3 is the only subunit of Pol δ that binds PCNA, using a nonconsensus PIP site that binds only one PCNA protomer of the trimeric ring. This structure has implications for the mechanism of clamp sliding during polymerase action, the possible role of the 4Fe-4S iron-sulfur cluster in Pol δ, the recycling of Pol δ during Okazaki fragment synthesis, and the ability of multiple factors to bind PCNA at the same time in the tool belt hypothesis.

### Mechanism of Sliding Clamps.

**Fig. 6.**
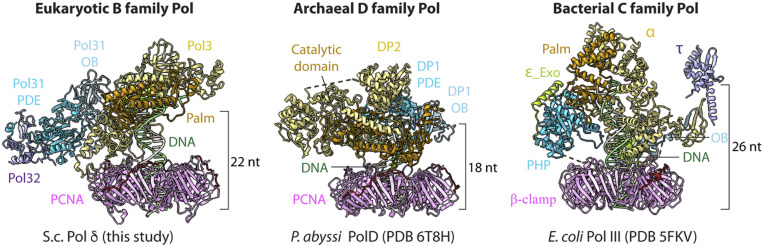
Comparison of replicative Pol-clamp-DNA structures of the three domains of life. In all three structures, the DNA is held by the Pol to thread through its respective clamp at a nearly perpendicular angle relative to the plane of the clamp. This is in sharp contrast to structures of PCNA and *E. coli* β clamps in the absence of a Pol, in which the DNA is angled sharply out of plane with the clamp (*SI Appendix*, Fig. S5*B*). Thus, the nearly perpendicular arrangement of DNA through the sliding clamps appears to generalize to replicases from the three domains of life. At this perpendicular orientation, and considering the large diameter of the clamp lumen, a layer of water will fit between the clamp and DNA. Thus, clamps appear to waterskate along duplex DNA from bacteria to human. The DNA length from the Pol active site to the bottom of the clamp ring varies among the Pol-clamp structure in the three domains of life. See the text for further details.

Structures of the eukaryotic PCNA clamp with DNA in the absence of Pol δ show that DNA is tilted by ∼28° with respect to the normal vector of the PCNA ring plane, such that DNA interacts directly with the α-helices that line the central lumen of the clamp (42, 43). DNA also passes through the *E. coli* clamp (in the absence of Pol) at a sharp angle and interacts directly with α-helices in the central chamber (42, 43); however, the tilt angle is much smaller (6°) when Pol δ is present (Fig. 6 and *SI Appendix*, Fig. S5). Thus, Pol δ holds the T/P DNA sufficiently tightly to position DNA straight through PCNA (Movie S1). The recently reported structures of *E. coli* Pol III-β clamp-DNA and archaeal Pol D-PCNA-DNA also show that the polymerase guides DNA nearly perpendicular to and straight through the ring (Fig. 6) (31, 32). It should be noted that the archaeal Pol D binds two PCNA subunits, unlike Pol δ, which binds only one PCNA subunit. The *E. coli* Pol III also binds the two subunits of the β clamp. Thus, while the nearly perpendicular angle of DNA through the clamp generalizes, the touchpoints of Pol-to-clamp do not.

The inner channel of all sliding clamps is 3 nm wide, much larger than needed to accommodate the 2-nm-wide dsDNA (4). Indeed, extension of positively charged side chains on the α-helices lining the inner chamber of PCNA do not reach within H-bonding distance to the phosphodiester backbone of DNA in the Pol δ-PCNA-DNA structure (Fig. 4*D* and Movie S1). Thus, there likely exists a layer of water molecules between PCNA and DNA in the Pol δ-PCNA-DNA complex, as originally suggested occurs for sliding clamps (2, 4). In other words, PCNA “waterskates” along DNA during synthesis while attached to Pol δ. Inspection of the bacterial and archaeal structures suggests to us that the concept of a clamp that waterskates, or “water planes,” on DNA during synthesis would appear to generalize. Therefore, we propose that the replicases of all three domains of life utilize a ring-shaped clamp that waterskates on dsDNA.

An obvious question arises whether there is any biological function for the eukaryotic PCNA and bacterial β clamps to tilt and physically contact DNA, in the absence of their corresponding DNA polymerase (*SI Appendix*, Fig. S5*B*) (42, 43). We suggest that the electrostatic interactions between clamp and DNA in the tilted clamp-DNA arrangement (in the absence of polymerase) provides “adherence” for the clamp to stay near the 3′ T/P junction after loading, where it can recruit and function with enzymes such as DNA polymerase, Fen1 nuclease, and ligase. Furthermore, we suggest that once the clamp loses the polymerase, it immediately switches from the perpendicular position to the tilted position with respect to the DNA, and that the electrostatic interaction with DNA hinders clamp sliding and keeps it close to the 3′ terminus for action with partner proteins.

### Possible Function of the 4Fe-4S Cluster in Sensing Intracellular Oxidation-Reduction State.

Pol δ is currently the only DNA polymerase structurally confirmed to have a 4Fe-4S cluster. The RNA primase subunit of DNA Pol α-primase—but not the Pol1 DNA Pol subunit—also contains a 4Fe-4S cluster (44). The 4Fe-4S cluster is coordinated differently in Pol δ and Pol α-primase, suggesting that the 4Fe-4S cluster plays a different role in these different enzymes (*SI Appendix*, Fig. S6*A*). Importantly, the 4Fe-4S cluster in CysB of Pol3 occupies a central location in Pol δ; it forms the interface between Pol3 and the Pol31-Pol32 regulatory subunits (Figs. 1*D* and 2 *A* and *B*), and it is adjacent to and interacts with the Pol3 CysA element that forms the interface with PCNA (Fig. 3*A*). Therefore, oxidation of the CysB 4Fe-4S cluster (Fe^2+^ to Fe^3+^) might alter protein conformation and thus biochemical activity, such as polymerase rate or processivity. In fact, oxidation of the 4Fe-4S cluster of Pol δ has been shown to slow the polymerase rate, suggesting that Pol δ may slow during oxidative stress when oxidative DNA damage occurs (20). Importantly, the oxidized 4Fe-4S cluster can be reversed (i.e., reduced) to regain activity (20). This suggests that the DNA replication apparatus—specifically, the Pol δ—may sense the intracellular oxidation-reduction state. Further work is needed to determine if this is truly the case.

#### **Potential roles of the regulatory subunits Pol31 and Pol32 in Pol δ function**.

##### Primase interaction (Pol32).

The Pol32 subunit contains a C-terminal canonical PIP site for PCNA, yet does not interact with PCNA in the structure. Interestingly, the replicative Pols of some bacteria and archaea also contain “unused” C-terminal PIP motifs; for example, the *E. coli* Pol III interacts with its clamp using an internal nonconsensus motif, such as S.c. Pol δ (33), but contains an unused C-terminal consensus motif (45). Likewise, the archaeal *Pyrococcus abyssi* replicative Pol D interacts with its clamp using an internal nonconsensus PIP motif (31) but contains a consensus C-terminal PIP motif. What do these “unused” consensus PIP motifs do? One obvious possibility is that unused PIP sites bind the clamp at some point during replication, so as to initially recruit the clamp or to retain a polymerase that has prematurely dissociated from its clamp. Below we suggest another possible role for these unused clamp-binding motifs.

*P. abyssi* Pol D was recently shown to use the C-terminal consensus PIP motif to bind the primase (32). Therefore, an intriguing possibility for the unused C-terminal consensus Pol32 PIP motif is to bind Pol α-primase. Supporting this suggestion, S.c. Pol32 has been demonstrated to interact with Pol α-primase (46, 47), and Pol31 was recently shown to help retain Pol δ in the replisome during multiple cycles of Okazaki fragment synthesis, supporting a connection between Pol32 and Pol α-primase during replisome function (48). Recent structural studies reveal that one Pol α-primase is anchored to the replisome by the Ctf4 trimer (49), and thus the Pol α-primase-Pol32 interaction likely recruits Pol δ to the replication fork. Despite these observations, however, whether Pol α-primase binds the PIP motif of Pol32 or some other region of Pol32 will require further study.

##### Polymerase recycling for multiple Okazaki fragments (Pol31).

We have shown in this study that Pol31-Pol32 binds ssDNA (*SI Appendix*, Fig. S3), possibly mediated by the OB and/or PDE domains of Pol31. The OB fold of Pol31 is ∼10 nt from the Pol3 active site. It should be noted that the ssDNA-binding activity has not been narrowed down to Pol31, because we could not purify it without Pol32. The ssDNA-binding activity of Pol31-Pol32 may have a role in the recycling of Pol δ for multiple lagging strand fragments by analogy to *E. coli* Pol III. *E. coli* Pol III rapidly recycles from a completed Okazaki fragment to a new primed site for the next Okazaki fragment, but the new primed site must contain a clamp (22, 23). Polymerase recycling during the synthesis of multiple Okazaki fragments, in which the Pol hops among clamps on newly primed sites, has been demonstrated for *E. coli*; this is often referred to as “collision release,” because it occurs at or soon after the Pol III collides with the 5′ terminus of the previous Okazaki fragment (50). Polymerase recycling in *E. coli* may be explained by recent structural studies. In the *E. coli* replisome, the ssDNA-binding domain of the tau subunit is positioned ahead of and laterally to Pol III (33). Therefore, it is proposed that when Pol III finishes an Okazaki fragment, the tau subunit has no ssDNA to grip, increasing the *K*_off_ of Pol III so that it can cycle to a new clamp on a new primed site near the fork (33).

S.c. Pol δ, like *E. coli* Pol III, undergoes rapid collision release from its PCNA clamp (21). Furthermore, we have recently shown by single-molecule methods that Pol δ extends numerous Okazaki fragments without dissociating from the replisome (48). The current structural study of Pol δ-PCNA-DNA reveals a further analogy to the *E. coli* system, in that the ssDNA-binding Pol31-Pol32 complex of Pol δ, like *E. coli* tau, is laterally displaced relative to the Pol3-PCNA-DNA axis. Thus, we suggest that Pol31-Pol32 may trigger collision release similar to that proposed for *E. coli* as illustrated in *SI Appendix*, Fig. S3.

##### Implications of the Pol δ-PCNA-DNA structure for the tool belt hypothesis.

**Fig. 7.**
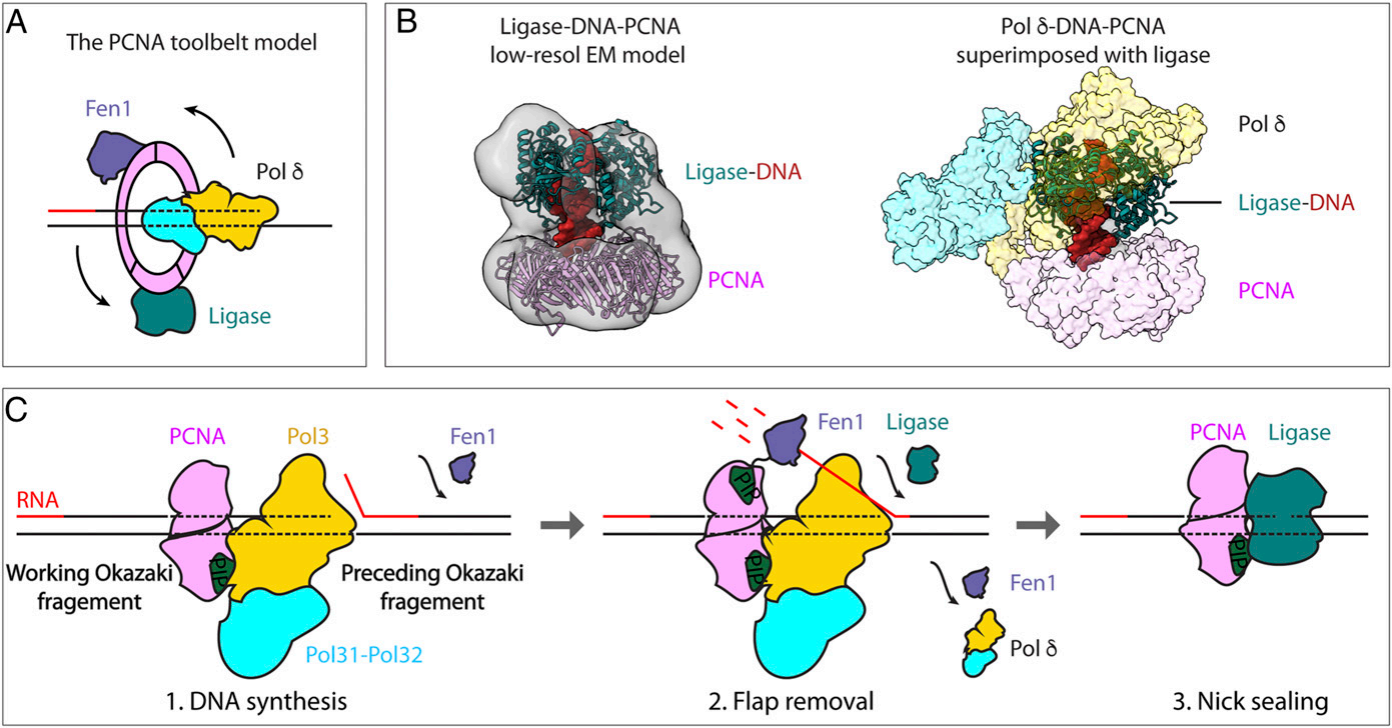
A model for Okazaki fragment synthesis. (*A*) The toolbelt model of Okazaki fragment synthesis suggests that Pol δ, Fen1, and ligase bind PCNA simultaneously. (*B*, *Left*) A 17-Å EM map (gray) of an archaeal DNA ligase-PCNA-DNA complex (48) docked with human ligase 1-DNA (PDB ID code 6P09) and yeast PCNA structure (PDB ID code 3K4X). (*B*, *Right*) Superimposition of the ligase-DNA-PCNA model with Pol δ-PCNA-DNA by overlapping the PCNA. Ligase sterically clashes with Pol δ. (*C*) A revised sequential switching model for Okazaki fragment synthesis. When it encounters an upstream RNA-DNA hybrid, Pol δ displaces the upstream 5′ end (step 1). The Fen1 5′ nuclease is recruited to Pol δ-PCNA for removal of the 5′ flap (step 2). On removal of RNA, ligase binds PCNA to seal the nick (step 3). The modeling in *B* suggests that Pol δ must be displaced for ligase to bind DNA-PCNA and accomplish the final step in Okazaki fragment maturation.

The bacterial β clamp is a homodimer and can bind the replicative Pol III and a TLS Pol at the same time (51). The hypothesis that the PCNA trimer may simultaneously bind the three enzymes used in Okazaki fragment synthesis/maturation (Pol δ, Fen1, and DNA ligase) is encompassed in the “tool belt” model, in which a homoligomer clamp binds more than one protein simultaneously (Fig. 7*A*) (52). As a prime example of the tool belt, the archaeal *S. solfataricus* PCNA is composed of three distinct gene products, each of which specifically binds either the replicative Pol, Fen1, or ligase (53). Furthermore, biochemical studies in yeast show that Pol δ and Fen1 coordinate their actions on a millisecond time scale (16, 31), and a recent structural study showed that human PCNA can accommodate Pol δ and Fen1 simultaneously (16, 31). However, assuming that the structure of Lig1-PCNA-DNA is the active form (Fig. 7*B*) (54), the Pol δ would be excluded due to steric occlusion, suggesting that the three enzymes might not bind the PCNA ring at the same time. Consistent with this scenario, the mutant PCNA trimer with only one functional binding site has been shown to support Okazaki fragment maturation (55). Therefore, the sequential switching of the partner enzymes, rather than simultaneous binding, may still explain PCNA-mediated Okazaki fragment synthesis/maturation (Fig. 7*C*). However, it remains possible that all three enzymes accommodate to one another on one PCNA clamp, and thus these queries will require further studies that include all three proteins in the same reaction.

## Materials and Methods

### Proteins and DNA.

S.c. Pol δ harboring the D321S and E323S mutations in the exonuclease domain of the catalytic subunit Pol3 (i.e., Pol δ^exo^-) was purified as described previously (*SI Appendix*, Fig. S3*C*) (11). To obtain the Pol31-Pol32 complex, genes encoding Pol31 and Pol32 were cloned into pCDF and pET11a and transformed into BL21(DE3)-competent *E. coli* cells. Cells were grown in 12 L of LB medium containing 50 μg/mL streptomycin and 100 μg/mL ampicillin at 37 °C to OD_600_ = 0.6, then rapidly lowered to 15 °C by swirling in an ice bath and induced with 0.2 mM isopropyl β-d-1-thiogalactopyranoside for 12 h at 15 °C before harvesting by centrifugation. Following lysis in a pressure cell and clarification by centrifugation, the GST-Pol31-Pol32 complex was purified on glutathione-Sepharose 4B (GE Healthcare). Then 5 mg of GST-Pol31-Pol32 was treated with GST-PreScission protease (GE Healthcare); dialyzed against 20 mM Tris⋅HCl pH 7.5, 0.1 mM EDTA, 5 mM DTT, and 100 mM NaCl; and further purified by passage over glutathione-Sepharose 4B to remove the GST tag and GST PreScission protease. Peak fractions were aliquoted and stored at −80 °C (*SI Appendix*, Fig. S3*C*). The T/P DNA was composed of template strand 5′-CTG​CAC​GAA​TTA​AGC​AAT​TCG​TAA​TCA​TGG-TCA​TAG​CT-3′ (38 nt) and primer strand 5′-AGCTATGACCATGATTACGAATTG-ddC-3′ (25 nt), which were synthesized and purified by Integrated DNA Technologies.

### Cryo-EM Grid Preparation and Data Collection.

To assemble the yeast Pol δ-PCNA-DNA complex, we first mixed 8.0 μM PCNA and 6.0 μM T/P DNA in 5.0 μL of reaction buffer containing 6.4 mM Hepes-KOH pH 7.5, 0.3 mM DTT, 16.3 mM MgAc, 8 mM KOAc, 8 mM potassium glutamate, and 0.4 mM ddTTP. Then 10 μL of 3.3 μM Pol δ was added. The final molar ratio of PCNA:Polδ:T/P DNA was 1.2:1:0.9. The reaction mixture was incubated on ice for 1 h. We glow-discharged the Quantifoil Cu R2/1 300 mesh grids for 1 min (Gatan Solarus), and applied 3 μL of sample on the EM grids. Sample vitrification was performed with the Vitrobot Mark IV system (Thermo Fisher Scientific) with the following settings: blot time 3 s, blot force 4, wait time 5 s, sample chamber temperature 6 °C, and 95% humidity. The EM grids were flash-frozen in liquid ethane cooled by liquid nitrogen. Cryo-EM data were automatically collected on a 300-kV Titian Krios electron microscope at the David Van Andel Advanced Cryo-Electron Microscopy Suite, using SerialEM. The EM images were formed at a scope magnification of 105,000× and with objective lens underfocus values ranging from 1.1 μm to 1.7 μm. Micrographs were recorded on a K3 direct electron detector (Gatan) operated in the superresolution video mode. During a 2-s exposure, a total of 40 frames were recorded with an accumulative dose of 68 e^−^/Å^2^. The calibrated pixel size was 0.414 Å for all digital micrographs.

### Image Processing and 3D Reconstruction.

We collected 12,559 raw micrographs during a 3-d session. The micrographs were subjected to a 5 × 5 patch alignment by MotionCor2 with a binning factor of 2 (56), and contrast transfer function estimation was performed with CTFFIND 4 (57) implemented in Relion 3.0 (58). Templates used for autopicking were generated by the human Pol δ-PCNA-DNA map (31) in cryoSPARC (59), and a total of 5,077,494 particles were picked. After two rounds of 2D image classification, the 2D classes of Pol δ alone or PCNA alone were discarded. This resulted in 428,820 particle images. These particles were used to yield four ab initio 3D models, and the particles belonging to the Pol δ-PCNA-DNA complex were further processed in cryoSPARC-v2 and Relion-3.0. The final 3D maps from cryoSPARC and Relion 3.0 were similar, with estimated resolutions of 3.2 Å and 3.1 Å, respectively. However, the 3.2-Å map from cryoSPARC had slightly better density at the Pol32 N-terminal region than the 3.1-Å map from Relion; thus, the 3.2-Å map was chosen as the final map. A typical raw image, selected 2D classes, and a brief flowchart of data processing are shown in *SI Appendix*, Fig. S1.

### Model Building, Refinement, and Validation.

To build the atomic model from the 3D map of the S.c. Pol δ-PCNA-DNA complex, we used three homologous structures: the yeast PCNA structure (Protein Data Bank [PDB] ID code 1SXJ), the yeast Pol δ structure (PDB ID code 6P1H), and the recently published human Pol δ structure (PDB ID code 6TNY). These atomic models were fitted into our 3D map. The PCNA atomic model was extracted from the S.c. RFC-PCNA complex structure (PDB ID code 1SXJ), the Pol δ atomic model was extracted from the S.c. Pol δ-DNA complex structure, and the DNA model was extracted from the human Pol δ structure (PDB ID code 6TNY), and these three parts were merged into one atomic model in UCSF ChimeraX (57). The merged model was adjusted by rigid body refinement in PHENIX (60) and then manually built in Coot (61). The CysA motif was disordered in the Pol δ-DNA structure without PCNA but was well ordered in our 3D map. This region was manually modeled based on the EM density. The manually built model was refined in real space in PHENIX. Owing to the flexibility of the two PCNA monomers that were not directly bound to Pol δ, we modeled these two PCNA protomers as a rigid body, and these protomers were not refined. The final model was refined to 3.2 Å and validated by MolProbity (62) as implemented in PHENIX validation. Structure figures were prepared using ChimeraX and organized in Adobe Illustrator.

### EMSA Assays.

EMSA assays were performed using either Pol31-Pol32 or Pol δ^exo-^ and a 5′-^32^P 62-mer (5′-ATG​CTT​AGC​CTG​AGG​ACT​ATT​CTA​CTT​AAC​GCG​AGT​TAC​GTG​ACG​GTA​TCT​TAT​CCG​GCG​C-3′). The Pol31-Pol32 complex (0 to 5 µM) or Pol δ^exo-^ (0 to 2.5 µM) was added to 0.5 nM ^32^P- 62mer in 50 mM Hepes (pH 7.5), 5 mM MgCl_2_, 50 mM NaOAc, 1 mM DTT, and 6% glycerol and incubated for 90 min at room temperature. Reactions were analyzed in 8% native polyacrylamide gel electrophoresis gels in 40 mM Tris and 150 mM glycine, pH 8.0. Gels were pressed dry and quantified using a Typhoon FLA 9500 scanner (GE Healthcare).

## Supplementary Material

Supplementary File

Supplementary File

## Data Availability

The 3D cryo-EM map of the S.c. Pol δ-PCNA-DNA-ddTTP complex at 3.2-Å resolution has been deposited in the Electron Microscopy Data Bank (ID code EMD-22803). The corresponding atomic model has been deposited in the PDB (ID code 7KC0). All other study data are included in the main text and *SI Appendix*.
