# Supplementary material for: Structure of eukaryotic DNA polymerase δ bound to the PCNA clamp while encircling DNA
Source: Proc Natl Acad Sci U S A. 2020 Nov 17;117(48):30344–53. doi: 10.1073/pnas.2017637117 (PMC7720213; doi:10.1073/pnas.2017637117)
Supplement: Supplementary File [file pnas.2017637117.sapp.pdf]

PNAS
www.pnas.org

Supplementary Information for

Structure of Eukaryotic DNA Polymerase δ Bound to the PCNA Clamp While Encircling DNA

By Zheng et al. Fengwei Zheng [1], Roxana E. Georgescu [2], Huilin Li [1,*], and Michael E. O'Donnell [2*]

[1] Structural Biology Program, Van Andel Institute, Grand Rapids, Michigan, USA
[2] DNA Replication Laboratory, The Rockefeller University and Howard Hughes Medical Institute, New York, New York, USA

[*]Correspondence should be addressed to H.L. (Huilin.Li@vai.org) or M.E.O. (odonnel@rockefeller.edu)

This PDF file includes:

Tables S1 and S2
Figures S1 to S7
Legend for Movie S1

Other supplementary materials for this manuscript include the following:

Movie S1

Supplementary Table 1. Cryo-EM 3D reconstruction and atomic model validation

	**Pol δ–PCNA–DNA**
**Data collection and processing**	
Magnification	105,000
Voltage (kV)	300
Electron dose (e^-/Å^2)	68
Under-focus range (μm)	1.1 – 1.7
Pixel size (Å)	0.828
Symmetry imposed	C1
Initial particle images (no.)	5,077,494
Final particle images (no.)	133,468
Map resolution (Å)	3.2
FSC threshold	0.143
Map resolution range (Å)	2.5 – 8.0
**Refinement**	
Initial model used (PDB code)	1SXJ, 6P1H, 6TNY
Map-sharpening B factor (Å^2)	-60.7
Map-to-model CC_{mask}	0.79
Model composition	
Non-hydrogen atoms	19,543
Protein and DNA residues	2,333, 47
Ligands	4
R.m.s. deviations	
Bond lengths (Å)	0.003
Bond angels (º)	0.558
Validation	
MolProbity score	2.24
Clashscore	10.23
Poor rotamers (%)	2.62
Ramachandran plot	

Favored (%)	94.12
Allowed (%)	5.88
Disallowed (%)	0

Supplementary Table 2. Disease-causing mutations in the human Pol δ and their correspondences in the S.c. Pol δ.

Human p125	Yeast Pol3	Location	Disease
E245K	D250	NTD	CRC, BDC
D316G/H	D321	Exo (active site)	CRC
E318K	E323	Exo (active site)	bGBM, NSCLC
R409W	R414	Exo	CRC
L474P	L479	Exo	CRC
S478N	S483	Exo	Colorectal adenoma/carcinoma
R506H	R511	Exo-Pol31_OB interface	CRC
R507C	R512[1]	Exo-Pol31_OB interface	MDPL
S605	S611	Palm	MDPL
L606M	L612	Palm	bPNET, bGBM, UCS
L632M	L638	Palm	bMDB, bGBM
R689W	R696	Fingers	LC, CRC, VSCC, bGBM, brain oligodendroglioma
V759I	V766	Palm	Colorectal adenoma/carcinoma
R1016H	K1013[2]	CysA-PCNA interface	EC, CRC, Brain glioma
R1060C	R1058	CysB- Pol31_OB interface	Immunodeficiency
I1070N	L1068[3]	CysB-Exo interface	MDPL
R1074W	K1072[4]	CysB-Exo interface	Autosomal-recessive syndrome
p50	Pol31		
D293N	D297	PDE	Autosomal-recessive syndrome

Abbreviations: CRC: Colorectal cancer, BDC: Bile duct cancer, bGBM: Brain glioblastoma, NSCLC: Non-small cell lung cancer, MDPL: Mandibular hypoplasia, deafness, progeroid features and lipodystrophy, EC: Endometrial cancer, LC: Lung cancer, VSCC: Vulvar squamous cell carcinoma, bMDB: Brain medulloblastoma, bPNET: Brain primitive neuroectodermal tumor, UCS: Uterine carcinosarcoma.

Underlined domains contain mutated residues.

[1]: Pol3 R512 is at the interface between Pol3 and Pol31 OB domain, but does not directly contact Pol31 in our structure.

[2]: CysA K1013 is at the interface between Pol3 and PCNA and forms a salt bridge with the PCNA D122 (see also **Fig. 2D**).

[3]: Pol3 L1068 is at the interface between the CysB and Exo domains, and does not directly contact the Exo domain in our structure. However, the distance between L1068 N and E492 OE2 in the Exo is close (4.1 Å).

[4]: Pol3 K1072 locates in the Pol3-Exo/Palm interface and does not contact Pol31 in our structure. However, the distances from K1072 NZ to T478 OG1 in Exo domain or to D925 OD2 in Palm domain are close, about 4 Å and 4.8 Å, respectively.

Supplementary Figures

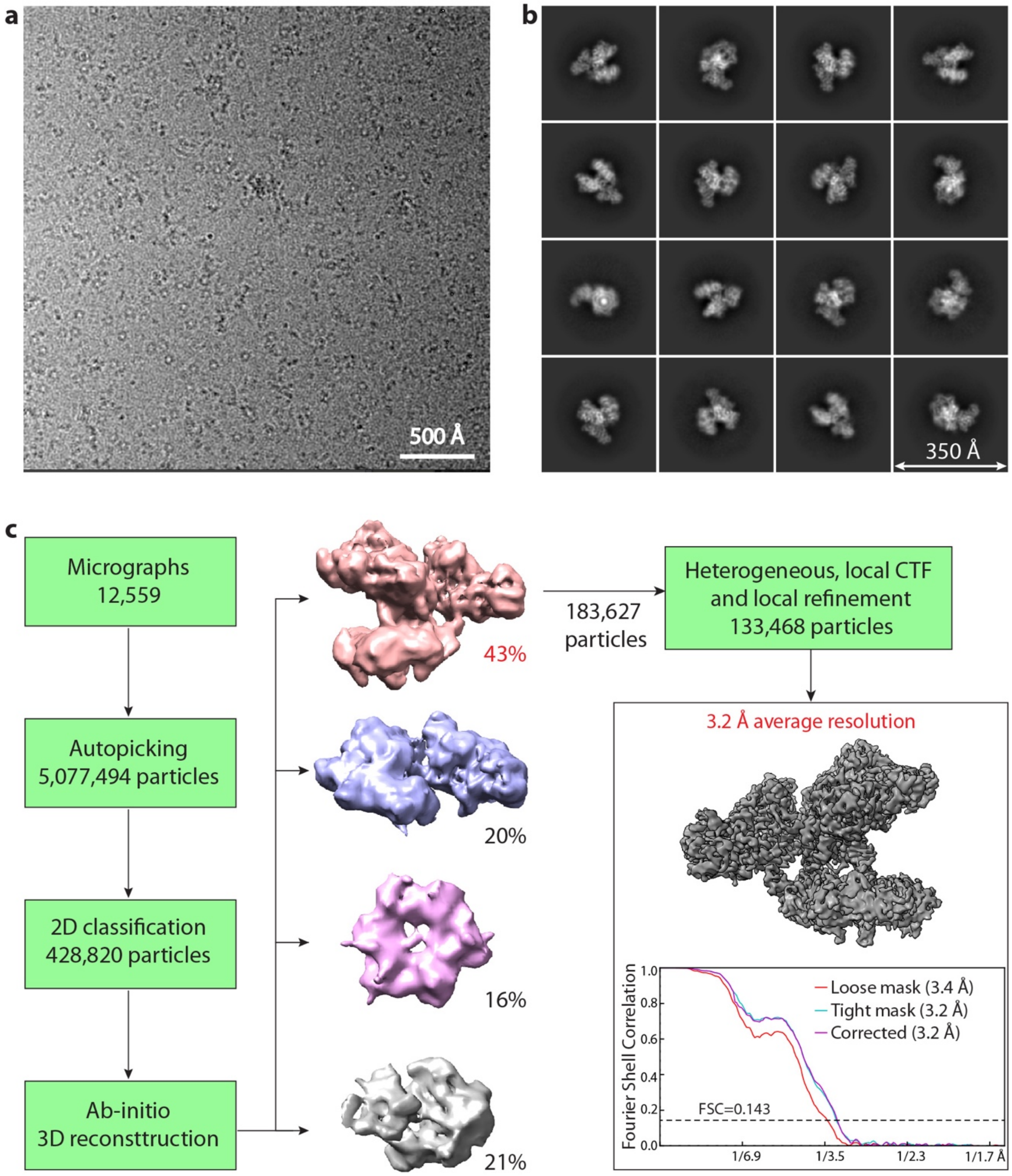

Supplementary Figure 1. Cryo-EM structure determination of S.c. Pol δ–PCNA–DNA. a) A representative raw micrograph recorded in Titan Krios on a K3 direct electron detector in super-resolution mode. **b**) Selected 2D class averages. **c)** The flowchart of data processing. The 0.143 criterion of the gold standard Fourier shell correlation was used to estimate the local resolution.

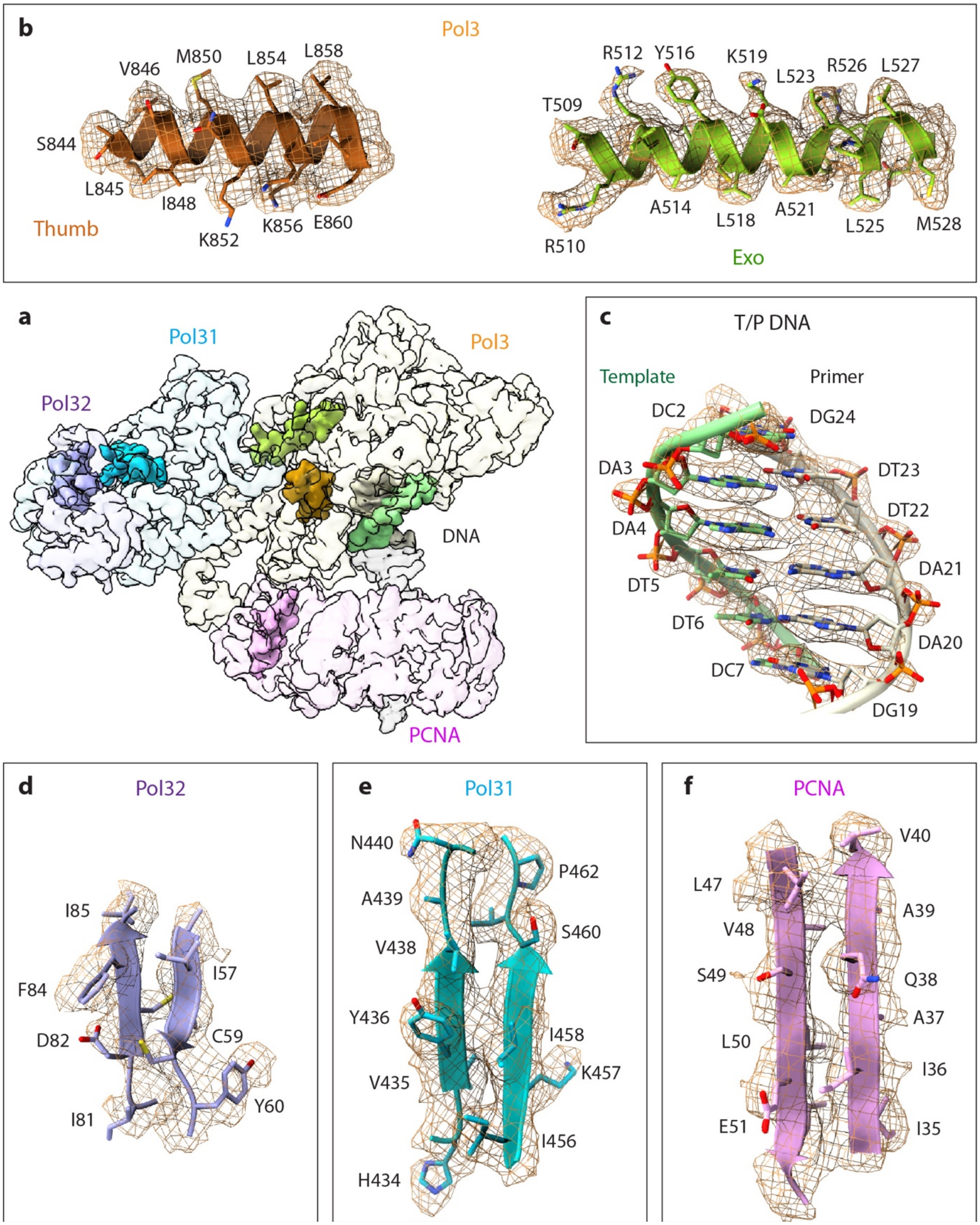

Supplementary Figure 2. Selected regions of the 3D map from each component of Pol δ–PCNA–DNA. a) Surface rendering of the cryo-EM map, The map is shown with 90% transparency except for the selected regions that are enlarged in panels b-f. **b-f**) Densities of the selected regions from catalytic Pol3 (b), T/P DNA (c), Pol32 (d), Pol31 (e), and PCNA (f).

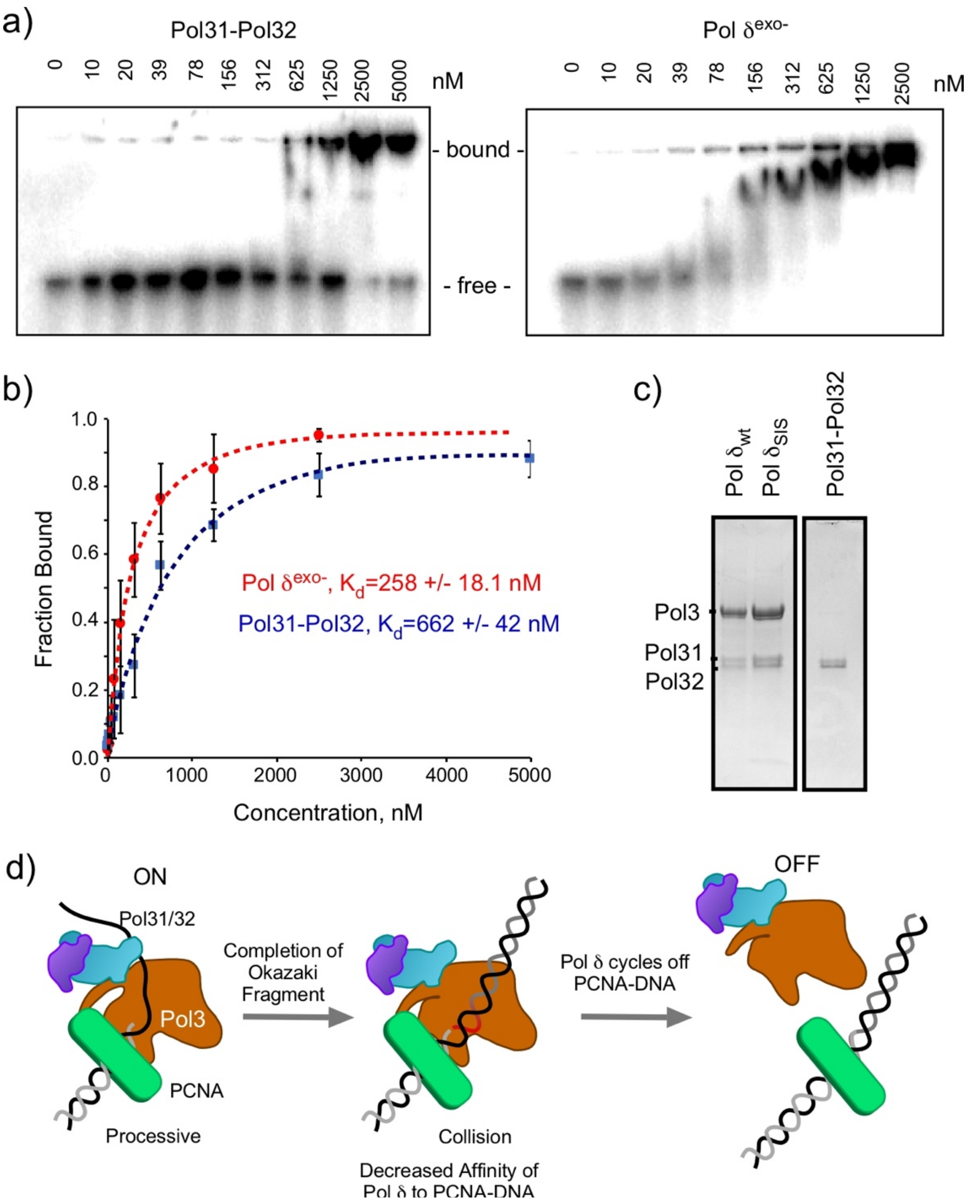

Supplementary Figure 3. Pol31-Pol32 interacts with ssDNA. **a)** Pol31-Pol32 complex or Pol δ^{exo-} were titrated into a reaction containing a 5'-^{32}P-62mer ssDNA, then analyzed for a mobility shift in a 8% native polyacrylamide gel. **b)** Quantitation of 4 replicates each of titrations of Pol31-Pol32 complex or Pol δ^{exo-} in separate EMSA analyses. **c**) SDS PAGE of protein preparations used in this work: wild type Pol δ, Pol δ^{exo-} and Pol31-Pol32 complex. **d)** Illustration of Pol δ recycling upon completing an Okazaki fragment. Left: Pol δ-PCNA is replicating primed DNA and Pol 31/32 binds template ssDNA, enhancing the grip of Pol δ to DNA. Middle: Pol δ finishes an Okazaki fragment upon colliding with the RNA primer, decreasing its affinity to PCNA-DNA. Right: During the lifetime of Pol δ on PCNA-DNA, it removes the primer with the assistance of Fen1 nuclease (not shown) but dissociates from PCNA-DNA soon afterward, freeing Pol δ to associate with a new PCNA clamp on the next primed site during lagging strand synthesis.

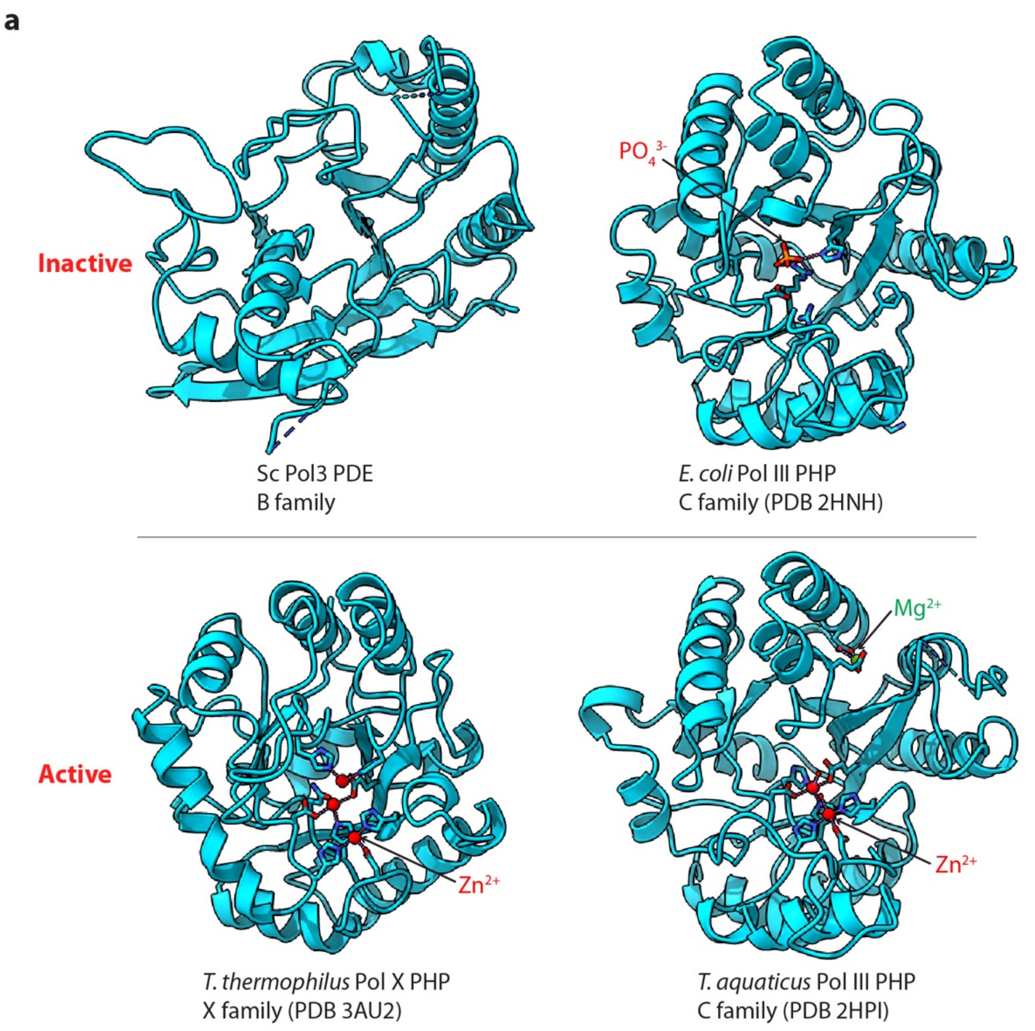

b

```
                 MBS       *  *            *              *             *
Taq Pol III_PHP    9 HLHQHTQFSL------LDGA 22    45 TDHG-N 49     70 GYEAY 74
Eco Pol III_PHP    8 HLRVHSDYSM------IDGL 21    42 TDFT-N 46     67 GADFN 71
Tth Pol X_PHP    340 DLQVHSTYS--------DGQ 351  372 TDHSP- 376   411 GAEVD 415
Sc  Pol31_PDE    232 RLEILREFLMGRINNKIDDI 251  272 NKDE-L 276   293 SISVD 297

                 MBS       *            *              *  *
Taq Pol III_PHP   93 YFHLT 97    143 SGCLG 147   210 TNDGHYV 216
Eco Pol III_PHP   81 LTHLT 85    132 SGGRM 136   199 TNDVRFI 205
Tth Pol X_PHP    434 LVLVS 438   477 RAPIE 481   527 STDAH-- 531
Sc  Pol31_PDE    314 PFHKS 318   369 PSNDN 373   430 DKWPHVY 436
```

Supplementary Figure 4. Comparison of the S.c. Pol31 PDE domain with its homologues from bacteria. a) Structures of active and inactive PDE/PHP domains. The active PDE/PHP domains contain 9 conserved metal binding sites (MBS) and adopt a barrel-shaped fold surrounded by several α-helixes. The *E. coli* Pol III PDE/PHP domain retains this folding but has lost 5 metal binding residues. The PHP/PDE domain of eukaryotes has a distorted barrel-shape and has lost 6 metal binding residues. **b**) Sequence alignment of the PDE/PHP domains shown in (a). The conserved MBS are marked by red asterisks.

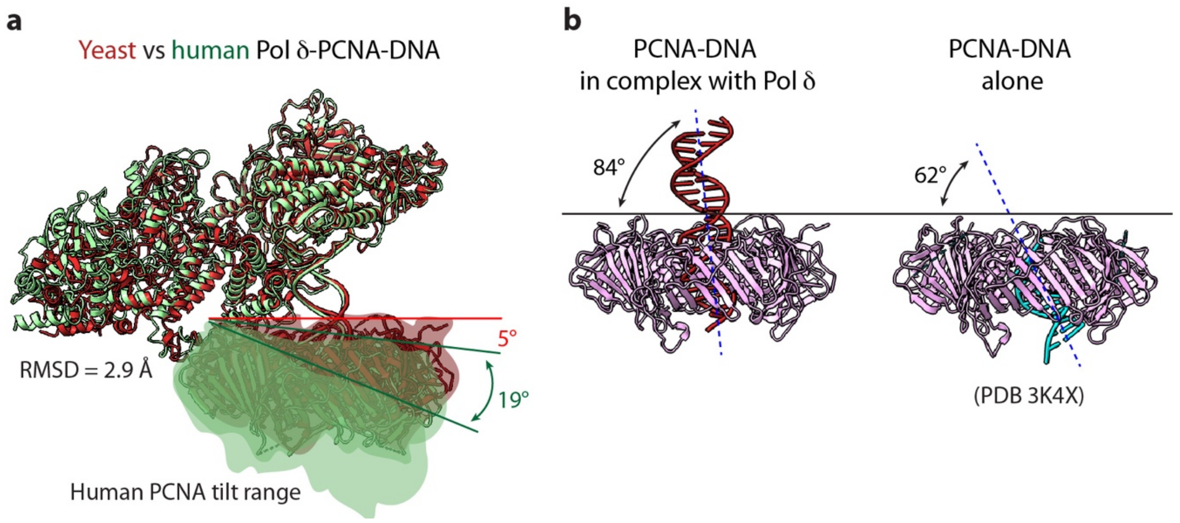

Supplementary Figure 5. Superimposition of human and S.c. Pol δ–PCNA–DNA and comparison to the DNA bound PCNA structure. a) Superimposition of S.c. Pol δ–PCNA (red) with the human Pol δ–PCNA structure in 3 configurations (green, PDB 6S1M, 6S1N and 6S1O). In the human structure, there were three conformers that differ in the tilt angle between PCNA and Pol δ; the most rigid human conformer 1 (PDB 6S1M) was 5° more tilted than in the S.c. structure. **b**) Comparison of the angle between DNA and PCNA in the S.c. Pol δ–PCNA–DNA structure (left) and in the human PCNA–DNA structure in the absence of Pol δ (PDB 3K4X).

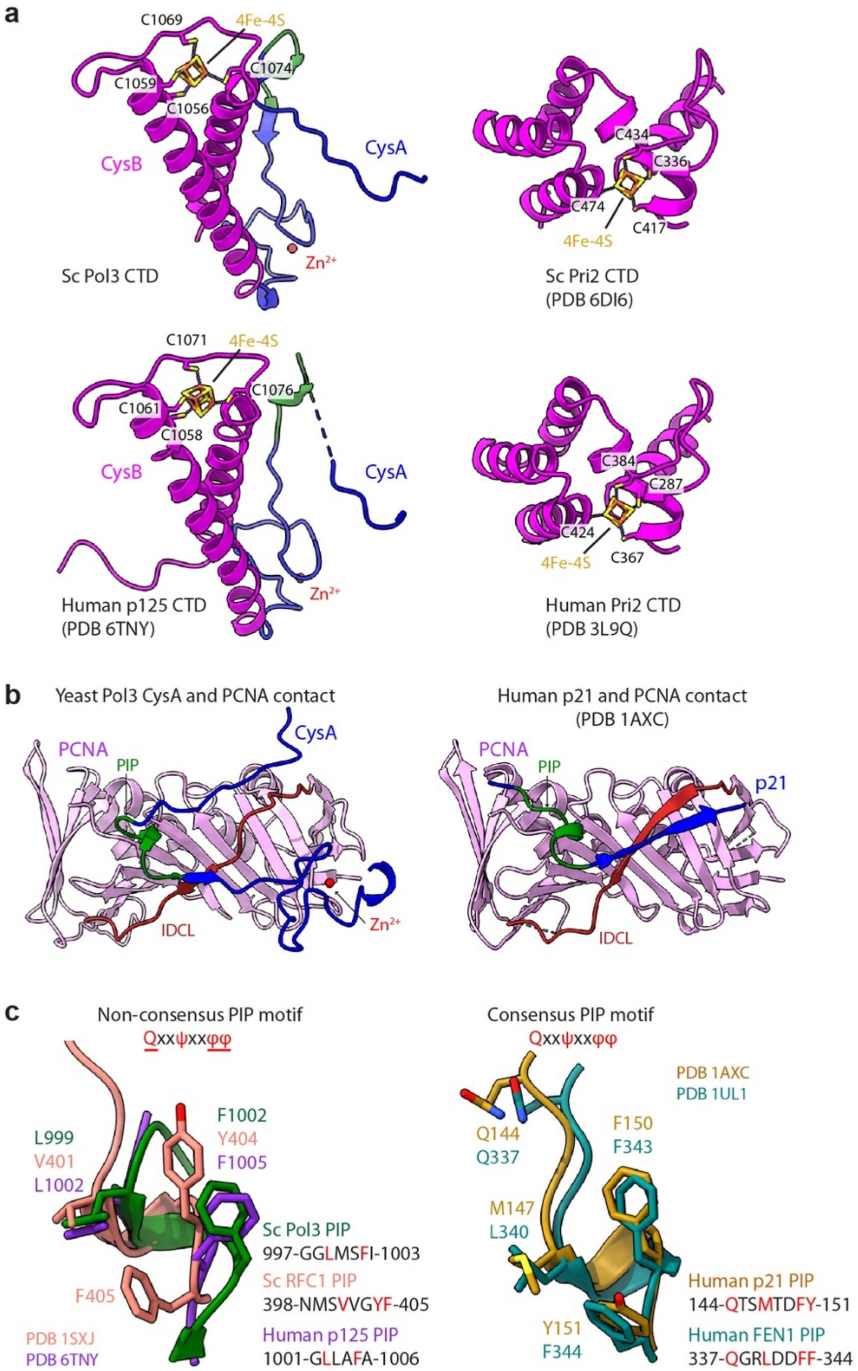

Supplementary Figure 6. Comparisons of consensus and non-consensus PIP motifs, their interactions with PCNA, and 4Fe-4S cluster coordination in Pol3 and Pol α-primase. **a**) Comparison of 4Fe-4S clusters in human and yeast Pol3 CysB elements with that in human and yeast Pri2 of Pol α-primase. Pol3 CysB consists of only two α-helices connected by a short loop. In contrast, the 4Fe-4S binding scaffolds of Pri2 are much larger (some regions are omitted in Pri2 for clarity). **b**) Comparison of Pol3 CysA versus p21 interactions with PCNA. p21 forms a longer antiparallel β–sheet than in Pol3 CysA. **c**) Comparison of non-consensus (left) vs consensus PIP motif (right) configurations.

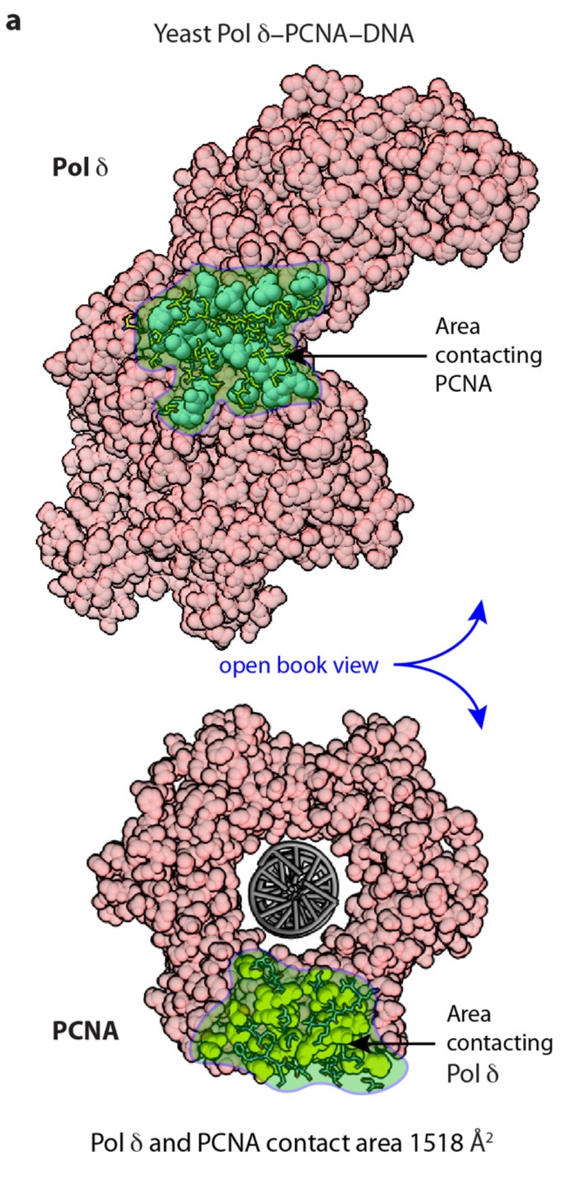

Supplementary Figure 7. Comparison of the Pol δ–PCNA interface in the yeast and human complexes. a) Open-book view of the yeast Pol δ–PCNA–DNA structure shown in salmon spheres. Contact surface in the yeast complex is 1518 $Å^2$. **b**) Open-book view of the human Pol δ–PCNA–DNA structure shown in light green spheres. Contact surface in the human complex is 1022 $Å^2$. The contact surface in yeast complex is 50% larger than in the human complex. In the upper panels of (**a-b**), residues from PCNA are shown in sticks. In the lower panels of (**a-b**), residues from Pol δ are shown in sticks.

Supplementary Movie 1. **3D perspective of S.c. Pol δ-PCNA-DNA**. Each Pol δ subunit, PCNA, and the DNA of the Pol δ-PCNA-DNA structure are shown in different colors, and rotated 360° in vertical and then horizontal planes. The structure is then positioned looking down the

DNA axis through the PCNA ring, and the PCNA-DNA component is than zoomed in to highlight the distance between DNA and the residues lining the central lumen of the PCNA clamp.
